# 2-Methyl-3-*p*-tolyl-3*H*-benzo[*e*]indole-1-carbonitrile

**DOI:** 10.1107/S1600536809052441

**Published:** 2009-12-12

**Authors:** Jiang-Sheng Li, Qi-Xi He, Peng-Yu Li

**Affiliations:** a School of Chemistry and Biological Engineering, Changsha University of Science & Technology, Changsha 410004, People’s Republic of China; b College of Chemistry and Chemical Engineering, Hunan University, Changsha 410082, People’s Republic of China

## Abstract

In the title compound, C_21_H_16_N_2_, the dihedral angle between the benzoindole and tosyl ring systems is 71.99 (7)°. In the crystal, mol­ecules are linked into centrosymmetric dimers by pairs of C—H⋯N hydrogen bonds, generating *R*_2_^2^(16) loops.

## Related literature

For the synthesis, see: Du *et al.* (2006 ▶).
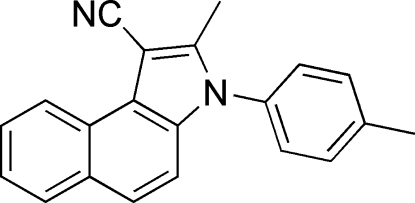


## Experimental

### 

#### Crystal data

C_21_H_16_N_2_
*M* *_r_* = 296.36
Monoclinic, 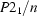

*a* = 10.321 (2) Å
*b* = 12.422 (3) Å
*c* = 13.258 (3) Å
β = 107.14 (3)°
*V* = 1624.4 (6) Å^3^
*Z* = 4
Mo *K*α radiation
μ = 0.07 mm^−1^
*T* = 293 K
0.28 × 0.20 × 0.18 mm

#### Data collection

Rigaku Saturn CCD diffractometer
Absorption correction: multi-scan (*CrystalClear*; Rigaku/MSC, 2005 ▶) *T*_min_ = 0.980, *T*_max_ = 0.987
10714 measured reflections
2858 independent reflections
2092 reflections with *I* > 2σ(*I*)
*R*_int_ = 0.036

#### Refinement

*R*[*F* ^2^ > 2σ(*F* ^2^)] = 0.056
*wR*(*F* ^2^) = 0.168
*S* = 1.04
2858 reflections
210 parameters
H-atom parameters constrained
Δρ_max_ = 0.19 e Å^−3^
Δρ_min_ = −0.16 e Å^−3^

### 

Data collection: *CrystalClear* (Rigaku/MSC, 2005 ▶); cell refinement: *CrystalClear*; data reduction: *CrystalClear*; program(s) used to solve structure: *SHELXS97* (Sheldrick, 2008 ▶); program(s) used to refine structure: *SHELXL97* (Sheldrick, 2008 ▶); molecular graphics: *SHELXTL* (Sheldrick, 2008 ▶); software used to prepare material for publication: *SHELXL97*.

## Supplementary Material

Crystal structure: contains datablocks I, global. DOI: 10.1107/S1600536809052441/hb5275sup1.cif

Structure factors: contains datablocks I. DOI: 10.1107/S1600536809052441/hb5275Isup2.hkl

Additional supplementary materials:  crystallographic information; 3D view; checkCIF report

## Figures and Tables

**Table 1:** Hydrogen-bond geometry (Å, °)

*D*—H⋯*A*	*D*—H	H⋯*A*	*D*⋯*A*	*D*—H⋯*A*
C16—H16⋯N2^i^	0.93	2.60	3.480 (3)	158

Symmetry code: (i) 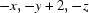.

**Fig. 1.**
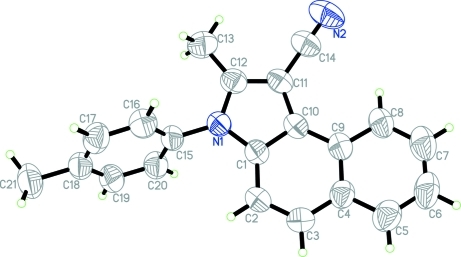
The molecule of (I) showing displacement ellipsoids drawn at the 50% probability level.

**Fig. 2.**
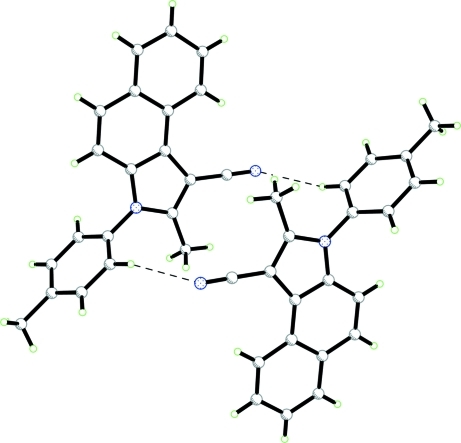
Centrosymmetric dimers of (I) formed by C—H···N hydrogen bonds drawn as dashed lines.

## supplementary crystallographic information

### Comment

The title compound, (I), comprises of a benzoindole ring and tosyl ring (Fig. 1). The dihedral angle of both rings is 71.99 (7)°.

In the crystal packing, molecules are linked into centrosymmetric dimers by C—H···N hydrogen bonds (Table 1). Weak C—H···π interactions help dimers pack. No significant π-π stacking interaction was observed.

### Experimental

The compound was obtained according to the method of Du and his coworkers (2006). Colourless block of (I) was grown by slow evaporation of its ethanolic solution.

### Refinement

All H atoms were positioned geometrically (C—H = 0.93 and 0.96 Å)and refined as riding with *U*_iso_(H) = 1.2*U*_eq_(CH) or 1.5*U*_eq_(CH_3_).

### Crystal data

**Table:**

C_21_H_16_N_2_	*F*(000) = 624
*M**_r_* = 296.36	*D*_x_ = 1.212 Mg m^−^^3^
Monoclinic, *P*2_1_/*n*	Mo *K*α radiation, λ = 0.71073 Å
Hall symbol: -P 2yn	Cell parameters from 3474 reflections
*a* = 10.321 (2) Å	θ = 2.2–27.5°
*b* = 12.422 (3) Å	µ = 0.07 mm^−^^1^
*c* = 13.258 (3) Å	*T* = 293 K
β = 107.14 (3)°	Block, colourless
*V* = 1624.4 (6) Å^3^	0.28 × 0.20 × 0.18 mm
*Z* = 4	

### Data collection

**Table:**

Rigaku Saturn CCD diffractometer	2858 independent reflections
Radiation source: rotating anode	2092 reflections with *I* > 2σ(*I*)
confocal	*R*_int_ = 0.036
Detector resolution: 7.31 pixels mm^-1^	θ_max_ = 25.0°, θ_min_ = 2.2°
ω and φ scans	*h* = −12→11
Absorption correction: multi-scan (*CrystalClear*; Rigaku/MSC, 2005)	*k* = −13→14
*T*_min_ = 0.980, *T*_max_ = 0.987	*l* = −15→14
10714 measured reflections	

### Refinement

**Table:**

Refinement on *F*^2^	Secondary atom site location: difference Fourier map
Least-squares matrix: full	Hydrogen site location: inferred from neighbouring sites
*R*[*F*^2^ > 2σ(*F*^2^)] = 0.056	H-atom parameters constrained
*wR*(*F*^2^) = 0.168	*w* = 1/[σ^2^(*F*_o_^2^) + (0.1027*P*)^2^] where *P* = (*F*_o_^2^ + 2*F*_c_^2^)/3
*S* = 1.04	(Δ/σ)_max_ = 0.001
2858 reflections	Δρ_max_ = 0.19 e Å^−^^3^
210 parameters	Δρ_min_ = −0.16 e Å^−^^3^
0 restraints	Extinction correction: *SHELXL97* (Sheldrick, 2008), Fc^*^=kFc[1+0.001xFc^2^λ^3^/sin(2θ)]^-1/4^
Primary atom site location: structure-invariant direct methods	Extinction coefficient: 0.070 (10)

### Special details

**Table:**

Geometry. All e.s.d.'s (except the e.s.d. in the dihedral angle between two l.s. planes) are estimated using the full covariance matrix. The cell e.s.d.'s are taken into account individually in the estimation of e.s.d.'s in distances, angles and torsion angles; correlations between e.s.d.'s in cell parameters are only used when they are defined by crystal symmetry. An approximate (isotropic) treatment of cell e.s.d.'s is used for estimating e.s.d.'s involving l.s. planes.
Refinement. Refinement of *F*^2^ against ALL reflections. The weighted *R*-factor *wR* and goodness of fit *S* are based on *F*^2^, conventional *R*-factors *R* are based on *F*, with *F* set to zero for negative *F*^2^. The threshold expression of *F*^2^ > σ(*F*^2^) is used only for calculating *R*-factors(gt) *etc*. and is not relevant to the choice of reflections for refinement. *R*-factors based on *F*^2^ are statistically about twice as large as those based on *F*, and *R*- factors based on ALL data will be even larger.

### Fractional atomic coordinates and isotropic or equivalent isotropic displacement parameters (Å^2^)

**Table:**

	*x*	*y*	*z*	*U*_iso_*/*U*_eq_	
N1	0.10089 (15)	0.87702 (12)	0.23973 (11)	0.0575 (5)	
N2	−0.2675 (2)	1.06928 (18)	0.01735 (16)	0.1025 (8)	
C1	0.00439 (18)	0.79674 (14)	0.23281 (13)	0.0547 (5)	
C2	0.0217 (2)	0.69469 (15)	0.27979 (14)	0.0629 (5)	
H2	0.1053	0.6728	0.3241	0.075*	
C3	−0.0883 (2)	0.62878 (16)	0.25826 (15)	0.0679 (6)	
H3	−0.0790	0.5606	0.2885	0.081*	
C4	−0.2171 (2)	0.66084 (17)	0.19091 (15)	0.0631 (6)	
C5	−0.3298 (2)	0.5901 (2)	0.16630 (16)	0.0806 (7)	
H5	−0.3197	0.5211	0.1947	0.097*	
C6	−0.4540 (2)	0.6220 (2)	0.10095 (19)	0.0900 (8)	
H6	−0.5266	0.5742	0.0854	0.108*	
C7	−0.4715 (2)	0.7253 (2)	0.05799 (18)	0.0860 (7)	
H7	−0.5561	0.7467	0.0150	0.103*	
C8	−0.3656 (2)	0.79452 (19)	0.07884 (15)	0.0692 (6)	
H8	−0.3783	0.8630	0.0491	0.083*	
C9	−0.23647 (19)	0.76498 (16)	0.14464 (13)	0.0580 (5)	
C10	−0.12000 (19)	0.83318 (14)	0.16682 (12)	0.0542 (5)	
C11	−0.0941 (2)	0.93897 (14)	0.13370 (13)	0.0589 (5)	
C12	0.0402 (2)	0.96343 (15)	0.17945 (14)	0.0608 (5)	
C13	0.1146 (3)	1.06418 (17)	0.17368 (17)	0.0812 (7)	
H13A	0.1546	1.0918	0.2436	0.122*	
H13B	0.0529	1.1164	0.1323	0.122*	
H13C	0.1847	1.0496	0.1414	0.122*	
C14	−0.1898 (2)	1.01126 (18)	0.06895 (15)	0.0722 (6)	
C15	0.24140 (19)	0.86902 (14)	0.29878 (14)	0.0556 (5)	
C16	0.3392 (2)	0.85881 (17)	0.24716 (15)	0.0670 (6)	
H16	0.3146	0.8569	0.1738	0.080*	
C17	0.4734 (2)	0.85143 (16)	0.30554 (17)	0.0722 (6)	
H17	0.5388	0.8442	0.2704	0.087*	
C18	0.5142 (2)	0.85442 (15)	0.41425 (17)	0.0670 (6)	
C19	0.4147 (2)	0.86356 (17)	0.46373 (16)	0.0717 (6)	
H19	0.4395	0.8647	0.5371	0.086*	
C20	0.2793 (2)	0.87108 (16)	0.40767 (14)	0.0661 (6)	
H20	0.2140	0.8775	0.4430	0.079*	
C21	0.6620 (2)	0.8465 (2)	0.4766 (2)	0.1000 (9)	
H21A	0.6952	0.7759	0.4682	0.150*	
H21B	0.6720	0.8594	0.5499	0.150*	
H21C	0.7128	0.8993	0.4513	0.150*	

### Atomic displacement parameters (Å^2^)

**Table:**

	*U*^11^	*U*^22^	*U*^33^	*U*^12^	*U*^13^	*U*^23^
N1	0.0540 (10)	0.0542 (9)	0.0598 (9)	0.0010 (7)	0.0098 (7)	0.0032 (7)
N2	0.1048 (18)	0.1026 (16)	0.0931 (13)	0.0401 (13)	0.0185 (12)	0.0307 (12)
C1	0.0532 (11)	0.0564 (11)	0.0521 (9)	0.0020 (9)	0.0115 (8)	0.0003 (8)
C2	0.0582 (12)	0.0630 (12)	0.0618 (11)	0.0065 (10)	0.0091 (9)	0.0085 (9)
C3	0.0706 (15)	0.0630 (12)	0.0686 (12)	−0.0020 (10)	0.0181 (10)	0.0123 (9)
C4	0.0561 (13)	0.0752 (14)	0.0595 (11)	−0.0083 (10)	0.0193 (9)	−0.0022 (9)
C5	0.0756 (17)	0.0909 (16)	0.0801 (13)	−0.0151 (13)	0.0306 (12)	−0.0002 (11)
C6	0.0581 (16)	0.124 (2)	0.0903 (16)	−0.0246 (14)	0.0249 (13)	−0.0114 (15)
C7	0.0544 (14)	0.123 (2)	0.0797 (14)	−0.0008 (13)	0.0185 (11)	−0.0047 (14)
C8	0.0516 (12)	0.0883 (15)	0.0654 (12)	0.0054 (11)	0.0137 (9)	−0.0024 (10)
C9	0.0517 (12)	0.0728 (13)	0.0502 (10)	0.0031 (9)	0.0162 (8)	−0.0029 (8)
C10	0.0531 (11)	0.0601 (11)	0.0475 (9)	0.0074 (9)	0.0120 (8)	−0.0010 (7)
C11	0.0616 (13)	0.0571 (11)	0.0550 (10)	0.0116 (9)	0.0128 (9)	0.0036 (8)
C12	0.0653 (13)	0.0532 (11)	0.0628 (10)	0.0061 (9)	0.0171 (9)	0.0013 (8)
C13	0.0884 (17)	0.0583 (13)	0.0960 (14)	−0.0013 (11)	0.0257 (13)	0.0075 (11)
C14	0.0755 (15)	0.0725 (14)	0.0667 (12)	0.0194 (11)	0.0181 (10)	0.0103 (10)
C15	0.0481 (11)	0.0550 (11)	0.0584 (10)	−0.0029 (8)	0.0078 (8)	−0.0001 (8)
C16	0.0665 (14)	0.0746 (13)	0.0614 (11)	0.0013 (10)	0.0213 (10)	−0.0024 (9)
C17	0.0578 (14)	0.0732 (14)	0.0902 (15)	0.0001 (10)	0.0288 (11)	−0.0033 (10)
C18	0.0535 (13)	0.0555 (12)	0.0852 (14)	−0.0010 (9)	0.0102 (11)	0.0016 (10)
C19	0.0628 (14)	0.0818 (14)	0.0627 (11)	−0.0043 (11)	0.0062 (10)	0.0005 (10)
C20	0.0564 (13)	0.0825 (14)	0.0593 (11)	−0.0021 (10)	0.0169 (9)	0.0014 (9)
C21	0.0563 (15)	0.0937 (18)	0.133 (2)	0.0003 (12)	0.0011 (14)	0.0023 (15)

### Geometric parameters (Å, °)

**Table:**

N1—C12	1.374 (2)	C10—C11	1.435 (3)
N1—C1	1.393 (2)	C11—C12	1.373 (3)
N1—C15	1.434 (2)	C11—C14	1.421 (3)
N2—C14	1.143 (2)	C12—C13	1.483 (3)
C1—C10	1.399 (2)	C13—H13A	0.9600
C1—C2	1.400 (2)	C13—H13B	0.9600
C2—C3	1.360 (3)	C13—H13C	0.9600
C2—H2	0.9300	C15—C20	1.380 (3)
C3—C4	1.422 (3)	C15—C16	1.382 (3)
C3—H3	0.9300	C16—C17	1.376 (3)
C4—C5	1.417 (3)	C16—H16	0.9300
C4—C9	1.420 (3)	C17—C18	1.378 (3)
C5—C6	1.377 (3)	C17—H17	0.9300
C5—H5	0.9300	C18—C19	1.376 (3)
C6—C7	1.394 (4)	C18—C21	1.509 (3)
C6—H6	0.9300	C19—C20	1.379 (3)
C7—C8	1.354 (3)	C19—H19	0.9300
C7—H7	0.9300	C20—H20	0.9300
C8—C9	1.409 (3)	C21—H21A	0.9600
C8—H8	0.9300	C21—H21B	0.9600
C9—C10	1.429 (3)	C21—H21C	0.9600
			
C12—N1—C1	109.04 (15)	C14—C11—C10	127.20 (19)
C12—N1—C15	125.88 (15)	C11—C12—N1	108.18 (17)
C1—N1—C15	125.07 (14)	C11—C12—C13	129.44 (18)
N1—C1—C10	108.42 (15)	N1—C12—C13	122.34 (18)
N1—C1—C2	128.59 (17)	C12—C13—H13A	109.5
C10—C1—C2	122.97 (17)	C12—C13—H13B	109.5
C3—C2—C1	117.55 (18)	H13A—C13—H13B	109.5
C3—C2—H2	121.2	C12—C13—H13C	109.5
C1—C2—H2	121.2	H13A—C13—H13C	109.5
C2—C3—C4	122.07 (18)	H13B—C13—H13C	109.5
C2—C3—H3	119.0	N2—C14—C11	179.4 (3)
C4—C3—H3	119.0	C20—C15—C16	119.78 (18)
C5—C4—C9	117.6 (2)	C20—C15—N1	119.91 (18)
C5—C4—C3	121.5 (2)	C16—C15—N1	120.31 (16)
C9—C4—C3	120.85 (18)	C17—C16—C15	119.24 (17)
C6—C5—C4	121.0 (2)	C17—C16—H16	120.4
C6—C5—H5	119.5	C15—C16—H16	120.4
C4—C5—H5	119.5	C16—C17—C18	122.2 (2)
C5—C6—C7	120.4 (2)	C16—C17—H17	118.9
C5—C6—H6	119.8	C18—C17—H17	118.9
C7—C6—H6	119.8	C19—C18—C17	117.41 (19)
C8—C7—C6	120.1 (2)	C19—C18—C21	121.3 (2)
C8—C7—H7	120.0	C17—C18—C21	121.2 (2)
C6—C7—H7	120.0	C18—C19—C20	121.91 (19)
C7—C8—C9	121.4 (2)	C18—C19—H19	119.0
C7—C8—H8	119.3	C20—C19—H19	119.0
C9—C8—H8	119.3	C19—C20—C15	119.5 (2)
C8—C9—C4	119.45 (19)	C19—C20—H20	120.3
C8—C9—C10	123.90 (19)	C15—C20—H20	120.3
C4—C9—C10	116.63 (17)	C18—C21—H21A	109.5
C1—C10—C9	119.89 (17)	C18—C21—H21B	109.4
C1—C10—C11	105.56 (17)	H21A—C21—H21B	109.5
C9—C10—C11	134.53 (16)	C18—C21—H21C	109.4
C12—C11—C14	123.93 (19)	H21A—C21—H21C	109.5
C12—C11—C10	108.80 (15)	H21B—C21—H21C	109.5
			
C12—N1—C1—C10	−0.03 (19)	C1—C10—C11—C12	−0.5 (2)
C15—N1—C1—C10	−178.86 (14)	C9—C10—C11—C12	−178.67 (19)
C12—N1—C1—C2	178.88 (18)	C1—C10—C11—C14	−177.42 (17)
C15—N1—C1—C2	0.0 (3)	C9—C10—C11—C14	4.4 (3)
N1—C1—C2—C3	−177.95 (18)	C14—C11—C12—N1	177.53 (16)
C10—C1—C2—C3	0.8 (3)	C10—C11—C12—N1	0.5 (2)
C1—C2—C3—C4	−0.1 (3)	C14—C11—C12—C13	0.0 (3)
C2—C3—C4—C5	177.9 (2)	C10—C11—C12—C13	−177.0 (2)
C2—C3—C4—C9	−1.2 (3)	C1—N1—C12—C11	−0.3 (2)
C9—C4—C5—C6	−0.8 (3)	C15—N1—C12—C11	178.55 (16)
C3—C4—C5—C6	−179.95 (19)	C1—N1—C12—C13	177.43 (18)
C4—C5—C6—C7	−0.4 (4)	C15—N1—C12—C13	−3.8 (3)
C5—C6—C7—C8	1.2 (4)	C12—C11—C14—N2	−129 (29)
C6—C7—C8—C9	−0.7 (3)	C10—C11—C14—N2	47 (29)
C7—C8—C9—C4	−0.6 (3)	C12—N1—C15—C20	110.1 (2)
C7—C8—C9—C10	177.95 (18)	C1—N1—C15—C20	−71.3 (2)
C5—C4—C9—C8	1.3 (3)	C12—N1—C15—C16	−70.1 (2)
C3—C4—C9—C8	−179.57 (17)	C1—N1—C15—C16	108.5 (2)
C5—C4—C9—C10	−177.31 (17)	C20—C15—C16—C17	−0.4 (3)
C3—C4—C9—C10	1.8 (3)	N1—C15—C16—C17	179.90 (17)
N1—C1—C10—C9	178.82 (15)	C15—C16—C17—C18	−0.3 (3)
C2—C1—C10—C9	−0.2 (3)	C16—C17—C18—C19	1.0 (3)
N1—C1—C10—C11	0.30 (19)	C16—C17—C18—C21	−179.8 (2)
C2—C1—C10—C11	−178.68 (16)	C17—C18—C19—C20	−0.9 (3)
C8—C9—C10—C1	−179.70 (15)	C21—C18—C19—C20	179.9 (2)
C4—C9—C10—C1	−1.1 (2)	C18—C19—C20—C15	0.3 (3)
C8—C9—C10—C11	−1.7 (3)	C16—C15—C20—C19	0.4 (3)
C4—C9—C10—C11	176.86 (18)	N1—C15—C20—C19	−179.86 (17)

### Hydrogen-bond geometry (Å, °)

**Table:**

*D*—H···*A*	*D*—H	H···*A*	*D*···*A*	*D*—H···*A*
C16—H16···N2^i^	0.93	2.60	3.480 (3)	158

Symmetry codes: (i) −*x*, −*y*+2, −*z*.
